# Unveiling a small new glassfrog of the genus *Vitreorana* (Anura, Centrolenidae) from Cerrado, central Brazil

**DOI:** 10.3897/zookeys.1291.193210

**Published:** 2026-09-03

**Authors:** Wilian Vaz-Silva, Sheila Pereira de Andrade, Cezar Filho Rocha, Edmar Pereira Victor Jr., Reuber Albuquerque Brandão, Natan Medeiros Maciel

**Affiliations:** 1 Pontifícia Universidade Católica de Goiás, Escola de Ciências Médicas e da Vida, Centro de Estudos e Pesquisas Biológicas; Programa de Pós-Graduação em Ciências Ambientais e Saúde, 74605-010, Goiânia, Goiás, Brazil Pontifícia Universidade Católica de Goiás, Escola de Ciências Médicas e da Vida, Centro de Estudos e Pesquisas Biológicas; Programa de Pós-Graduação em Ciências Ambientais e Saúde Goiânia Brazil; 2 Universidade Federal de Goiás, Instituto de Ciências Biológicas, Departamento de Ecologia, Laboratório de Herpetologia e Comportamento Animal; Programa de Pós-graduação em Biodiversidade Animal, Campus Samambaia, 74690-900, Goiânia, Goiás, Brazil Universidade Federal de Goiás, Instituto de Ciências Biológicas, Departamento de Ecologia, Laboratório de Herpetologia e Comportamento Animal; Programa de Pós-graduação em Biodiversidade Animal, Campus Samambaia Goiânia Brazil; 3 Centro Tecnológico de Engenharia, Rua 254, 146, Setor Coimbra, 74.535-440, Goiânia, Goiás, Brazil Centro Tecnológico de Engenharia Goiânia Brazil; 4 Universidade de Brasília, Departamento de Engenharia Florestal, Laboratório de Fauna e Unidades de Conservação, 70910-900, Brasília, Distrito Federal, Brazil Universidade de Brasília, Departamento de Engenharia Florestal, Laboratório de Fauna e Unidades de Conservação Brasília Brazil

**Keywords:** Amphibia, bioacoustics, genetic distance, glassfrog, integrative taxonomy

## Abstract

A new species of the glassfrog genus *Vitreorana* is described from Central Brazil based on an integrative approach combining morphological, molecular, and bioacoustic data. *Vitreorana micaelae***sp. nov**. is distinguished by the combination of the following characters: small body size (21.7–26.1 mm); parietal and visceral peritonea bearing white iridophores; snout shape rounded in dorsal and slopping in profile; presence of cloacal ornamentation; and advertisement call features composed of two call types (short and long calls). The new species represents the second known glassfrog endemic to the Cerrado biome, being associated with humid gallery forests. Uncorrected p-distances for the 16S rRNA gene fragment among sampled *Vitreorana* species range from 3.5% to 9.6%, with the new species showing the lowest divergence with *V. parvula*.

## Introduction

Anurans of the genus *Vitreorana* Guayasamin, Castroviejo-Fisher, Trueb, Ayarzagüena, Rada & Vilà, 2009 are commonly known as glassfrogs and are primarily characterized by the presence of a white hepatic peritoneum covering the liver, a white gastrointestinal peritoneum, and green bones (Guayasamin et al. 2009). Species of *Vitreorana* mostly occur in both montane and lowland regions of northern South America, including northern Venezuela, the Guiana Shield, the Amazon Basin, and the Atlantic Forest, typically at elevations below 1900 m.

Currently, the genus includes ten species (Zucchetti et al. 2023; Frost 2026): *Vitreorana parvula* (Boulenger, 1895), *V. eurygnatha* (Lutz, 1925), *V. ritae* (Lutz, 1952), *V. antisthenesi* (Goin, 1963), *V. helenae* (Ayarzagüena, 1992), *V. gorzulae* (Ayarzagüena, 1992), *V. castroviejoi* (Ayarzagüena & Señaris, 1997), *V. baliomma* Pontes, Caramaschi & Pombal, 2014, *V. franciscana* Santana, Barros, Pontes & Feio, 2015, and *V. assuh* Zucchetti, Rojas-Padilla, Dias, Solé, Orrico & Castroviejo-Fisher, 2023.

Notably, *Vitreorana franciscana* occurs within the Cerrado biome, where it is associated with semideciduous forest and gallery forest. This contrasts with the other species in the genus that are typically found in the Amazonia and Atlantic Forest (Santana et al. 2015; Maffei et al. 2024). Additionally, some records of *V. eurygnatha* also exhibit this occurrence pattern, occasionally being syntopic with *V. franciscana* (Bang et al. 2020).

Despite records of *Vitreorana* in marginal areas of the Cerrado, the first report of the genus for the core region of the Cerrado, in central Brazil, was made by Cintra et al. (2013) based on a single individual tentatively identified as *V. eurygnatha*. This identification was not followed by Vaz-Silva et al. (2020), who considered the specimen as an undescribed species.

Recently, we reanalyzed the specimen reported by Cintra et al. (2013) and analyzed further collected individuals of *Vitreorana* glassfrogs from a new locality in the central region of the Cerrado in the state of Goiás, Brazil. We found that morphological, bioacoustic, and molecular evidence support the recognition of these populations as a distinct new species. Herein, we provide a formal description, discuss its diagnostic features and also geographic distribution.

## Materials and methods

### Morphological data

We examined specimens deposited in the herpetological collections of the Pontifícia Universidade Católica de Goiás – PUC Goiás, Goiânia, Brazil (**CEPB**), and the Museu Nacional, Rio de Janeiro, Brazil (**MNRJ**). All examined specimens are listed in Suppl. material 1. The type series is deposited in the herpetological collection of Pontifícia Universidade Católica de Goiás (**CEPB**).

Morphological terminology follows Cisneros-Heredia and McDiarmid (2007) and Guayasamin et al. (2020). Comparisons with the other species were based on direct examination of specimens, original descriptions, and diagnoses provided by Zucchetti et al. (2023). Internal characters were assessed through dissection of a paratype (CEPB 10156), following the terminology of Cisneros-Heredia and McDiarmid (2007). Webbing formulae follow Savage and Heyer (1997), as modified by Guayasamin et al. (2006). The humeral structure was examined to assess the presence of humeral spines in males by dissecting the ventrolateral region of the arm of a paratype (CEPB 10158). Color in life was described based on photographs of live individuals. External morphometric variables follow Zucchetti and Castroviejo-Fisher (2024). Twenty measurements were taken using Mitutoyo digital calipers (± 0.01 mm) under a Zeiss Stemi DV4 stereomicroscope, as follows. The description of coloration in life and in preservative was based on individuals of the type series.

**SVL** Snout–Vent Length;

**HL** Head Length;

**HW** Head Width;

**IOD** Interorbital Distance;

**UE** Upper Eyelid Width;

**IN** Internarial Distance;

**EN** Eye-Nostril Distance;

**SE** Snout-Eye Distance;

**ED** Horizontal Eye Diameter;

**TD** Tympanum Diameter;

**ET** Eye-Tympanum Distance;

**RUL** Radio-Ulna Length;

**HDL** Hand Length;

**F2L** Finger-II Length;

**F3L** Finger-III Length;

**4DW** Disc of Finger-IV;

**F4W** Finger-IV Width;

**TL** Tibia Length;

**THL** Thigh Length;

**FL** Foot Length.

### Bioacoustics data

We analyzed 41 advertisement calls from three individuals recorded at the type locality on 5 December 2014, between 20:00 and 23:00 h, air temperature of 20 °C. Recordings were obtained using a Marantz PMD 671 digital recorder (44.1 kHz sampling rate, 16-bit resolution) coupled with a Sennheiser ME66/K6 directional microphone. Acoustic analyses were conducted in Raven Pro 1.6.5 (64-bit; Cornell Lab of Ornithology 2022) using the following settings: window type = Hanning; window size = 256 samples; 3 dB filter bandwidth = 270 Hz; brightness = 75%; contrast = 65%; overlap = 85% (locked); DFT size = 1,024 samples; and grid spacing (spectral resolution) = 46.9 Hz. Figures were generated using the packages seewave v. 2.2.3 (Sueur et al. 2008) and soundshape (Rocha and Romano 2021) in R v. 4.3.3 (R Development Core Team 2024). All recordings are deposited in the Fonoteca at the Coleção Zoológica (FONO–UFG 1802, FONO–UFG 1803, and FONO–UFG 1804) of the Universidade Federal de Goiás (UFG), Goiânia, Goiás, Brazil.

Data are presented as mean ± standard deviation and range. The analyses were based on a call-centered approach. Call terminology follows Köhler et al. (2017). The following call parameters were analyzed: call duration; pulse number by notes; note rate; and dominant frequency. Advertisement call parameters were compared with published descriptions for other species of *Vitreorana* (Haga et al. 2014; Zaracho 2014; Santana et al. 2015; Bang et al. 2020; Zucchetti and Castroviejo-Fisher 2024).

### Genetic data

Hepatic tissue samples from the holotype (CEPB 10154) and one paratype (CEPB 10157) were collected and preserved in 70% ethanol. A fragment of the mitochondrial 16S rRNA gene (primers: 16sar: CGCCTGTTTAT-CAAAAACAT and 16sbr: CCGGTCTGAACTCAGATCACGT) (Palumbi et al. 1991), was amplified and sequenced to assess genetic distances. Amplification was performed using standard PCR protocols (Vences et al. 2005) with an annealing temperature of 60 °C. PCR products were purified using Exonuclease I and Shrimp Alkaline Phosphatase. Sequencing was conducted in ABI 3500 Genetic Analyzer; Applied Biosystems, Inc. USA in both forward and reverse directions, and consensus sequences were generated in SeqScape 2.7 (Applied Biosystems). Sequences were edited in CodonCode Aligner v. 9.0.2 (CodonCode Co., USA) and aligned using MAFFT v. 7 with E-INS-I (strategy Katoh and Standley 2013).

A dataset comprising 553 bp of the 16S rRNA gene for nine species (eleven terminals, being two sequences of *V. parvula* and the new species and the other species with only one sequence) of *Vitreorana*, was assembled to assess genetic distances among taxa (Suppl. material 2). Uncorrected pairwise genetic distances (p-distances) were calculated in MEGA with pairwise deletion of gaps (Tamura et al. 2021). Sequences of *V. assuh* and *V. franciscana* were not available; therefore, genetic distances between these species and other congeners, including the new species, could not be assessed.

## Results

### Vitreorana micaelae sp. nov.

Taxon classificationAnimaliaAnuraCentrolenidae

7EEDD5EA-4FED-5594-9EB0-4DEB6FFF62A8

https://zoobank.org/44105CE3-9E22-4E27-B4E2-DC7F5E82024E

Vitreorana eurygnatha —Cintra et al. 2013: 587–590.
Vitreorana sp.—Vaz-Silva et al. 2020: 32.

#### Type material.

**Figure 1.**
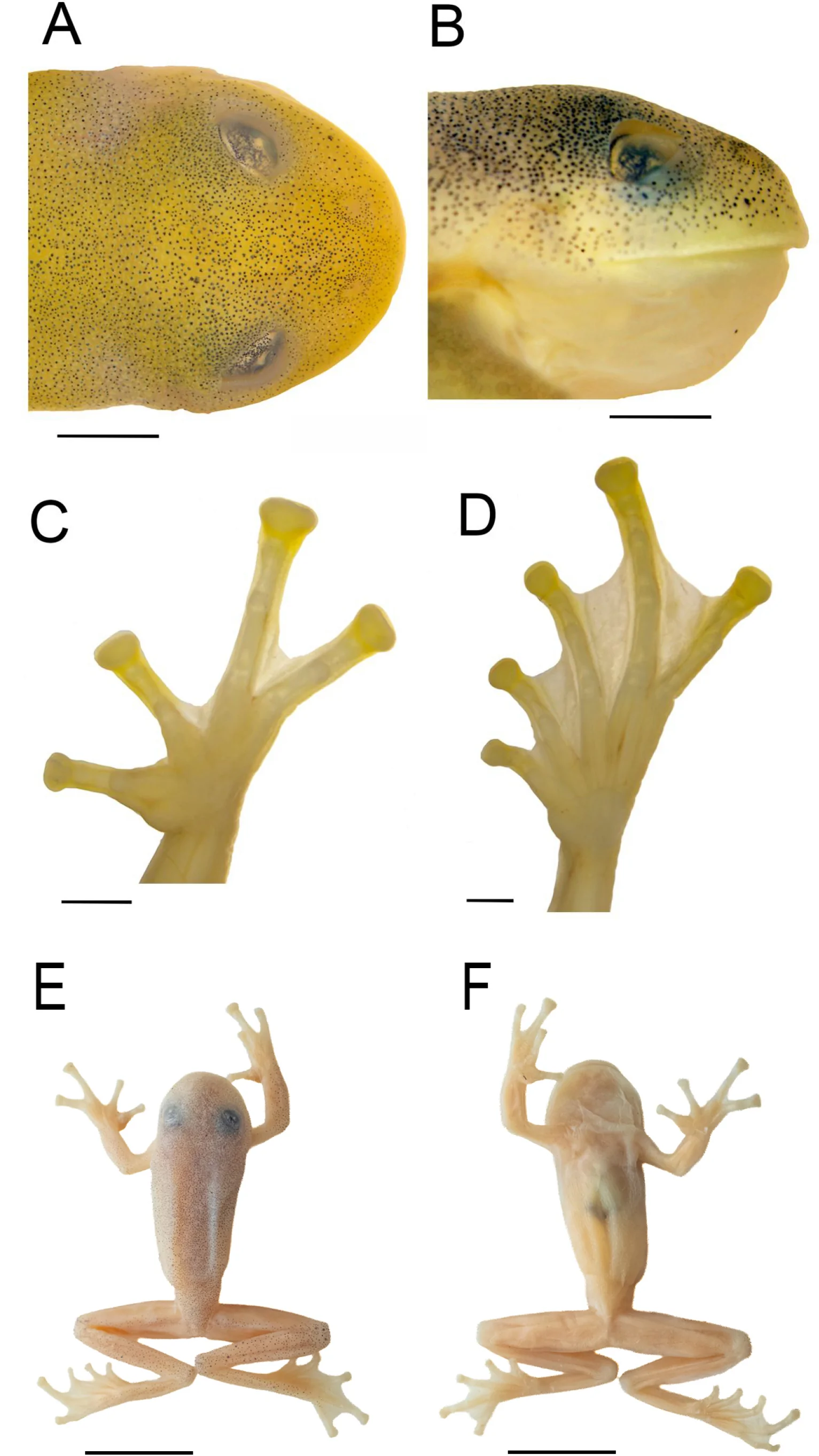
*Vitreorana micaelae* sp. nov. **A**. Dorsal; **B**. Lateral views of the head; **C**. Palmar; **D**. Plantar views; **E**. Dorsal; **F**. Ventral views of the holotype (CEPB 10154). Scale bars: 3 mm (**A–D**); 10 mm (**E, F**).

***Holotype***. • CEPB 10154 (Fig. 1), an adult male from the municipality of Luziânia, state of Goiás, Brazil, collected in a gallery forest along a small stream near the east bank of the São Bartolomeu River (16°06.87'S, 47°43.50'W; 826 m a.s.l.), on 9 December 2014 by Cezar Filho Rocha. ***Paratypes***. • Eight adult males, all specimens were collected at the type locality by Cezar F. Rocha and Edmar P. Victor Jr: • CEPB 10151, collected on 5 December 2014; • CEPB 10152, collected on 6 December 2014; CEPB 10153, collected on 8 December 2014; and CEPB 10155–10159, collected with the holotype on 9 December 2014. Additionally, an adult male CEPB 7712 collected during the faunal rescue operation owing to the filling of the Corumbá IV Hydroelectric Power Plant reservoir, in the municipality of Luziânia, state of Goiás, on 4 July 2005.

#### Diagnosis.

**Figure 2.**
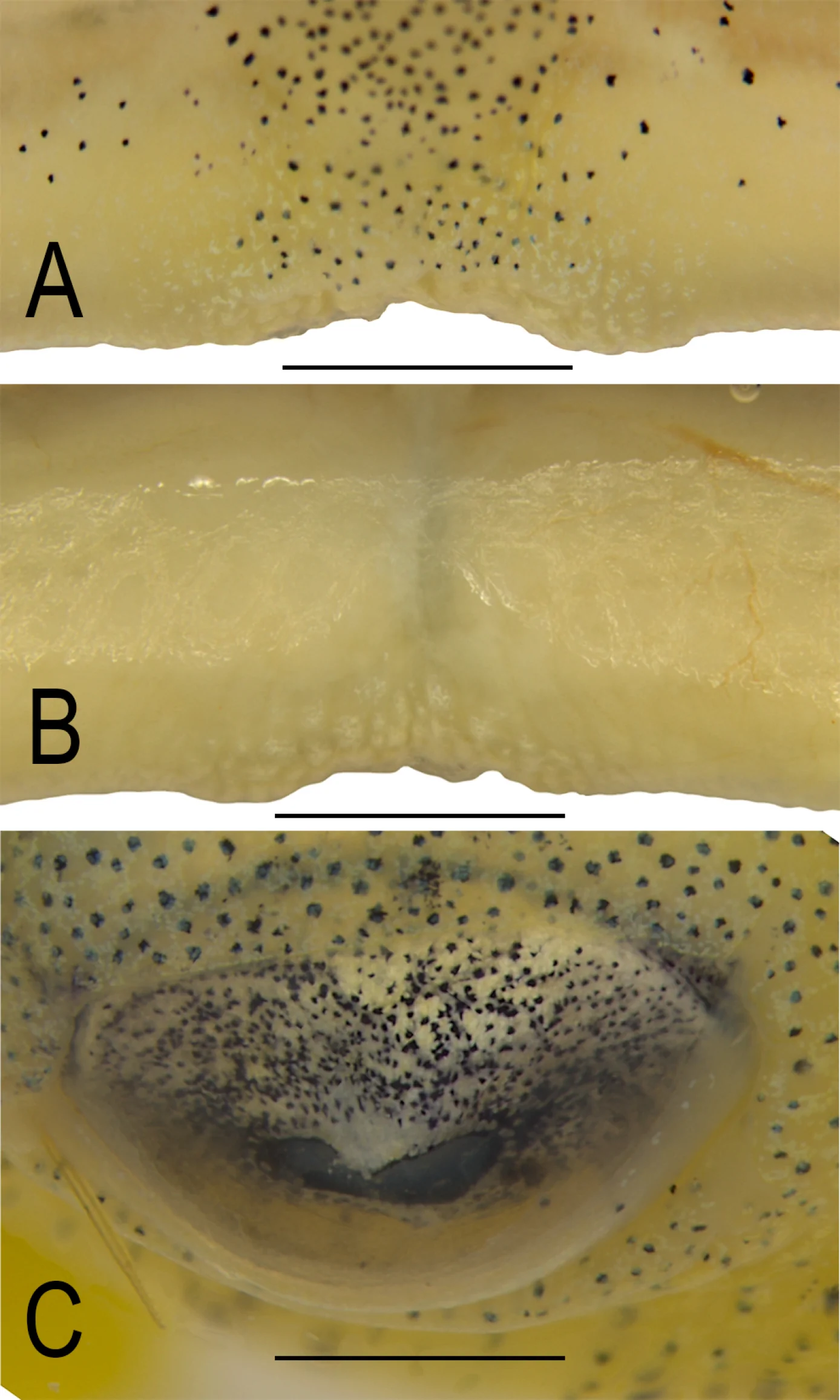
Cloacal ornamentation in **A**. Dorsal; **B**. Ventral views (CEPB10154, holotype); **C**. Iris detail with dark flecks in preservative (CEPB10157, paratype) in *Vitreorana micaelae* sp. nov. Scale bars: 1.5 mm.

*Vitreorana micaelae* sp. nov. is characterized by the following combination of characters: 1) small body size (SVL 23.2 ± 1.2 mm in males, ranging from 21.7–26.1 mm); 2) vomerine teeth and dentigerous process of vomers absent; 3) tympanum evident, colored as the surrounding skin, with diameter ~ 33% of the eye diameter; 4) tympanic annulus barely visible; 4) dorsal skin texture shagreen; 5) snout rounded in dorsal view and sloping in profile; 8) cloacal ornamentation composed of small, enamel-like tubercles of different sizes, increasing in size toward the venter (Fig. 2); 9) parietal peritoneum translucent (P0 condition); 10) iridophores covering the pericardium, hepatic peritoneum, and urinary bladder peritoneum; all other visceral peritonea transparent (V4 condition); 11) bulbous liver (H2 condition); 12) webbing absent between fingers II and III, reduced between fingers III and IV, and IV and V, with webbing formula: III 2–3^1/2^ IV 2^1/2^–2^+^ V; (13) webbing between toes moderate, webbing formula: I 1^+^–2^+^ II 1^+^–2^1/3^ III 1^+^–2^+^ IV 2^+^–1^+^ V; 14) humeral crest and spine absent in adult males; 15) dorsal coloration in life leaf green with minute black punctuations; 16) melanophores forming punctuations regularly distributed on dorsum; 17) in preservative, the dorsum is cream with abundant dark melanophore punctuations; hands and feet bear few melanophores; the outer surface of the arm has a lower concentration of melanophores than the forearm; and the upper eyelids show a higher concentration of melanophores; 18) iris whitish cream with dark flecks; 19) discs on fingers III and IV broadly enlarged and truncate; 20) advertisement call composed of two calls types containing 1–4 notes, 2–3 pulses by notes, call duration ranges from 17.1–25.1 ms (call type I) and 45.6–115.6 ms (call type II) with dominant frequency ranging 4651.2–4737.3 Hz (call type I) and 4694.2–4758.8 Hz (call type II).

#### Comparison with other species of the genus.

Features of previously described species are presented within parenthesis. *Vitreorana micaelae* sp. nov. differs from *V. helenae* by its larger size, and from *V. assuh* by its smaller size. *Vitreorana micaelae* sp. nov. has a snout-vent length ranging from 21.7–26.1 mm in adult males (vs 18.9–20.4 mm in *V. helenae*; and 30.9–34.1 mm in *V. assuh* (see Ayarzagüena 1992; Señaris and Ayarzagüena 2005; Kok and Castroviejo-Fisher 2008; Zucchetti et al. 2023). In *Vitreorana micaelae* sp. nov., the dorsal skin is shagreened (vs smooth in *V. baliomma*, and *V. eurygnatha*). The new species also differs from *V. franciscana*, *V. baliomma*, and *V. eurygnatha*) by its rounded snout in dorsal view (vs subovoid snout in *V. franciscana* and truncate snout in *V. baliomma* and *V. eurygnatha*). The snout profile is sloping in *Vitreorana micaelae* sp. nov. (vs snout rounded in *V. baliomma*, truncated or rounded in *V. eurygnatha*, and slightly truncate in *V. parvula*). The parietal peritoneum is translucent in *Vitreorana micaelae* sp. nov. (P0 condition based in Cisneros-Heredia and McDiarmid 2007) (vs parietal peritoneum partially covered, P2 condition, in *V. ritae*, *V. antisthenesi*, *V. helenae*, *V. gorzulae*, and *V. castroviejoi*). In the new species, the visceral peritoneum presents iridophores covering the pericardium, hepatic peritoneum, and urinary bladder peritonea. In contrast, all other parts of the visceral peritoneum are clear (V4 condition) (vs V5 condition in *V. ritae*, *V. baliomma*, and *V. gorzulae*). *Vitreorana micaelae* sp. nov. has a bulbous liver (H2 condition) (vs lobed in *V. ritae* and *V. castroviejoi*, H1 condition). In the new species, cloacal ornamentation is composed of small, enamel-like tubercles of varying sizes, becoming larger toward the venter and bearing white pigmentation (Fig. 2) (vs presence of small tubercules similar in size in *V. helenae* and *V. ballioma*; cloacal ornamentation formed by a more developed granular structure in *V. parvula*). Additionally, *Vitreorana micaelae* sp. nov. differs from other species of *Vitreorana* by the advertisement call with emission pattern with two call types (single call in other species) (Table 2).

#### Description of the holotype.

Adult male (CEPB 10154), with a single, subgular vocal sac. Body slender; head distinct, wider than long; HL 28.3% and HW 34.8% of SVL; *canthus rostralis* indistinct; loreal region slightly concave; nostrils ovoid and protuberant, closer to tip of snout, directed dorsolaterally; internarial area barely depressed; moderate-sized eyes, ED 40% of F3L, directed anterolaterally; ED narrower than IOD (ED/IOD = 49%); transverse diameter of disc of Finger IV 52.3% of the eye diameter. Tympanum of small size (TD 42.8% of ED). Vomerine teeth absent; choanae large and rounded; tongue ovoid, ventral posterior half not attached to the floor of mouth; vocal slits extending posterolaterally from the lateral edge of the tongue to the angle of the jaw. Upper lip bearing a supralabial white line.

Arm slender, forearms slightly thicker than arms with minute and star-shaped melanophores; relative finger lengths I <II < IV < III; finger discs expanded, elliptical and truncate on the distal margin; subarticular tubercles small and rounded; supernumerary tubercles present; palmar tubercle elliptical, elongated. Webbing absent between fingers II and III, reduced between fingers III and IV, and IV and V, with webbing formula: III 2–3^1/2^ IV 2^1/2^–2^+^ V.

Slender hind limbs, FL 70.8% and TL 53.2% of the SVL; relative toe length I < II < III ≈ V < IV; toe discs expanded, elliptical, and truncated on the distal margin; webbing between toes moderate, webbing formula: I 1^+^–2^+^ II 1^+^–2^1/3^ III 1^+^–2^+^ IV 2^+^–1^+^ V; inner metatarsal tubercle large, ovoid, near the base of toe I; subarticular tubercles small and rounded; supernumerary tubercles present. Dorsal surfaces shagreened; gular region and ventral surface of forelimbs smooth; venter and ventral surface of the hindlimbs areolate with small and round granules, all similar in size. Ventral surfaces and flanks cream. Cloacal ornamentation composed of enamel-like tubercles of different sizes, increasing in size toward the venter.

***Measurements of the holotype* (mm)**. SVL 26.1; HL 7.4; HW 9.1; IOD 4.2; UE 0.9; IN 1.8; EN 2.2; SE 3.9; ED 2.1; TD 0.9; ET 0.9; RUL 5.2; HDL 7.8; F2L 3.5; F3L 5.2; 4DW 1.1; F4W 0.8; TL 13.9; THL 12.5; FL 18.5.

**Figure 3.**
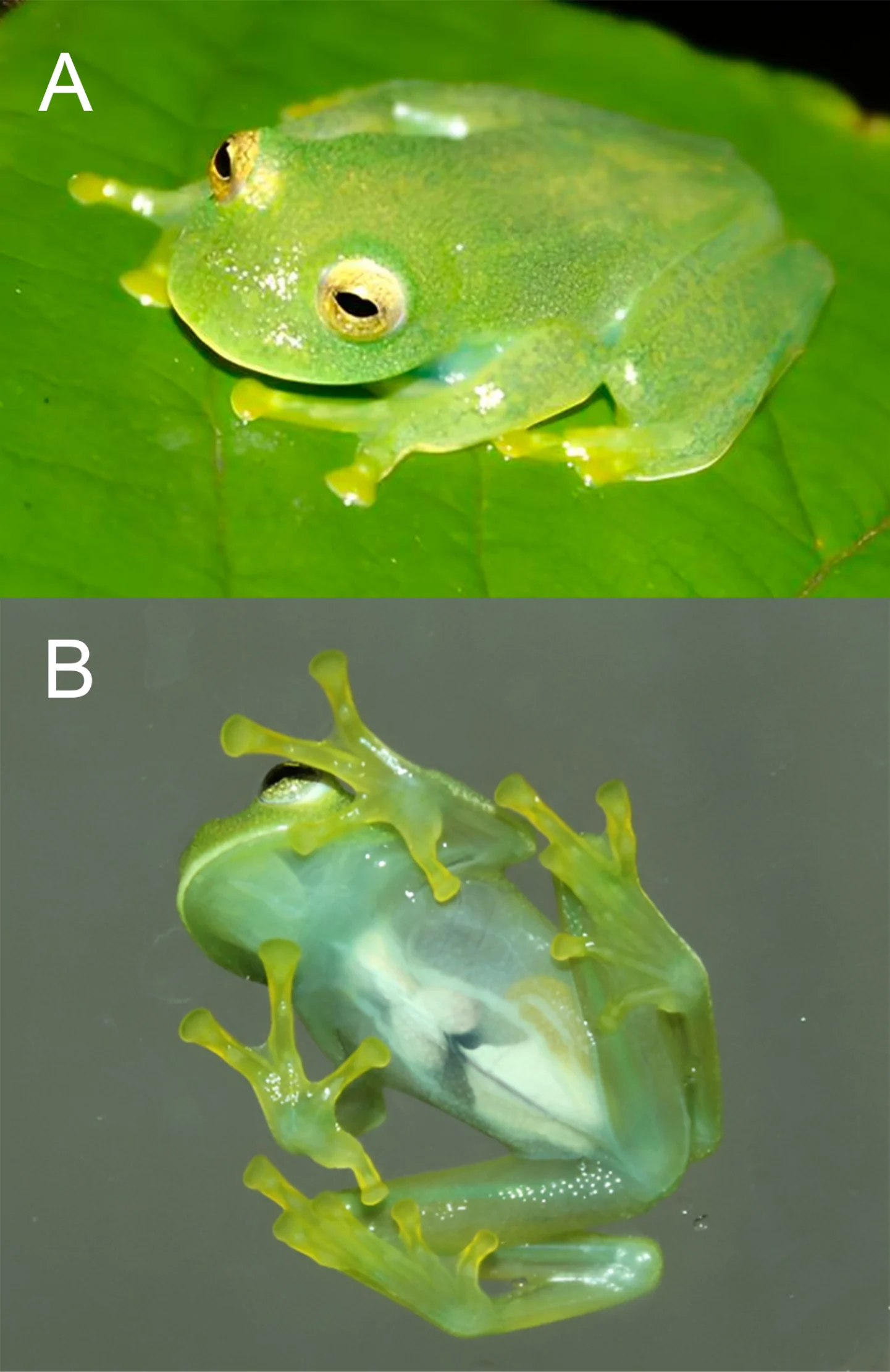
Color patterns in live specimens of *Vitreorana micaelae* sp. nov. from the type locality in Luziânia, State of Goiás, Brazil. **A, B**. CEPB10157. Photos by C.F. Rocha.

***Color in life***. Dorsal coloration light green, with yellow tones on the flanks and extremities of the limbs and digits. Minute black melanophores distributed throughout the dorsal surface and limbs. Flanks without melanophores. Paler green ring surrounding the eye. Upper lip bearing a supralabial white line. Ventral surface of the limbs, hands, and feet greenish. Venter translucent, evidencing the white visceral peritoneum with white iridophores (Fig. 3).

***Color in preservative***. In preservative, arms, hands, legs, feet, dorsum, and venter whitish cream, with dark melanophores in star-shaped or asterisk-shaped regularly distributed.

***Variation***. Variation was observed in the dorsal pattern in the distribution of melanophores. Specimen CEPB10156 shows a higher concentration of melanophores on the left side of the head, forming a patch, and with blotchy aspects in the region of the articulation of the arm with the forearm and of the thigh with the tibia. Specimens CEPB10157, CEPB10159, and CEPB10155 showed little variation in the arrangement of melanophores. On the other hand, specimen CEPB10159 showed a subtle increase in concentration in the frontoparietal region of the head. Specimen CEPB10155 showed an increase in spots in the elbow, knee, and frontoparietal regions. Specimens CEPB7712, CEPB10153, and CEPB10154 presents a dorsal pattern with smaller and less clustered melanophores forming punctuations, resulting in a less reticulated pattern. The variation in morphometric measurements is presented in Table 1.

**Table 1.** Measurements (mm) of ten males of *Vitreorana micaelae* sp. nov., nine from the type-locality (CEPB 10151–CEPB 10159) and the specimen CEPB 7712 (Cintra et al. 2013).

**Character**	**Mean ± SD (mm)**	**Range (mm)**
SVL	23.2 ± 1.2	21.7–26.1
HL	7.3 ± 0.3	7.0–7.8
HW	8.3 ± 0.5	7.4–9.1
IOD	3.7 ± 0.5	2.9–4.3
UE	1.0 ± 0.2	0.8–1.6
IN	1.6 ± 0.3	1.1–2.3
EN	1.8 ± 0.3	1.2–2.2
SE	3.1 ± 0.4	2.4–3.9
ED	2.1 ± 0.2	1.7–2.3
TD	0.7 ± 0.1	0.6–0.9
ET	1.2 ± 0.3	0.9–1.9
RUL	4.9 ± 0.2	4.7–5.2
HDL	7.8 ± 0.3	7.5–8.4
F2L	3.4 ± 0.3	2.9–3.7
F3L	5.2 ± 0.3	4.8–5.7
4DW	1.3 ± 0.3	0.7–1.5
F4W	0.9 ± 0.1	0.7–1.1
TL	13.3 ± 0.6	12.7–14.6
THL	12.8 ± 0.7	12.0–14.2
FL	17.6 ± 0.9	16.7–19.5

#### Bioacoustics.

**Figure 4.**
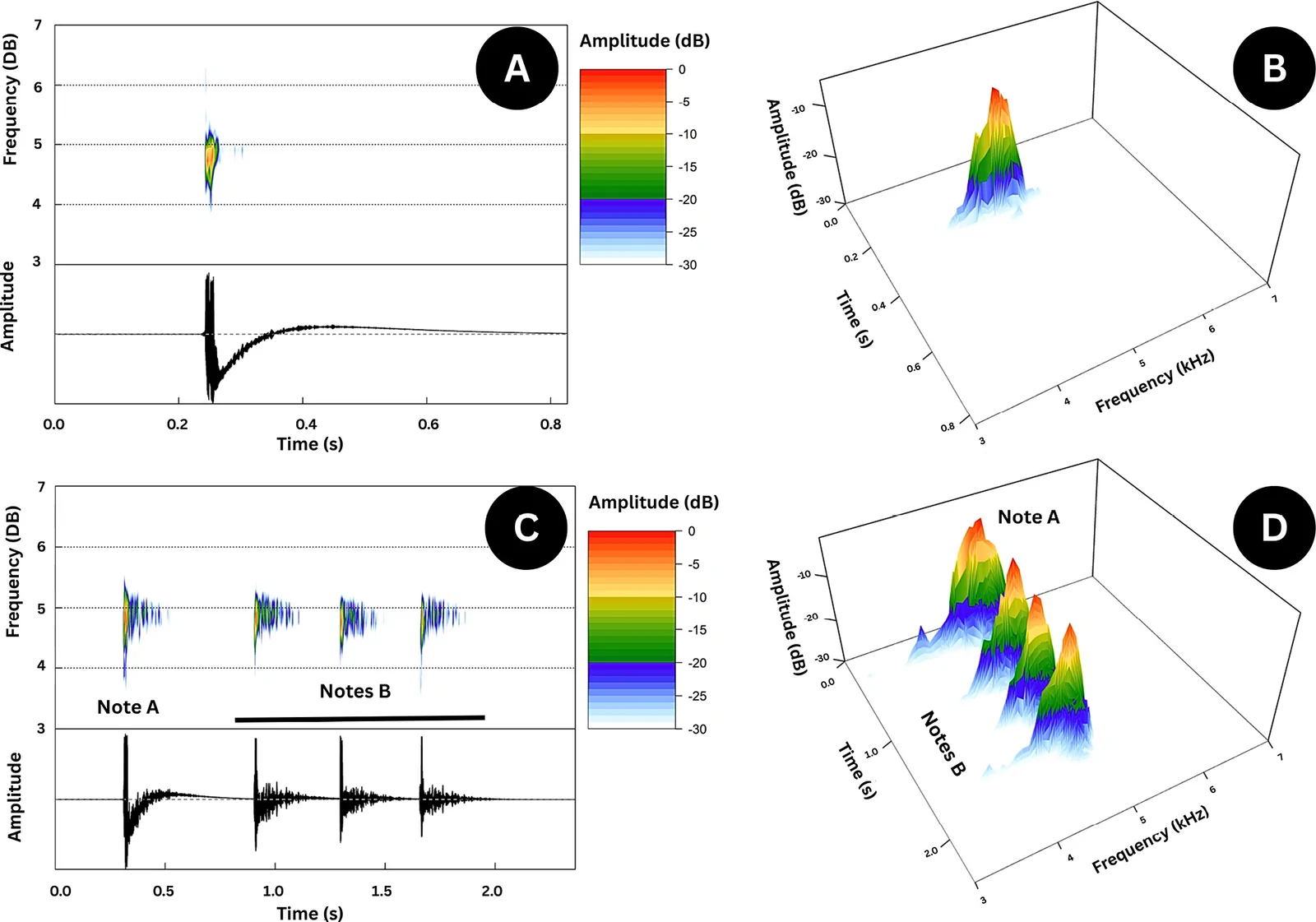
Spectrograms and oscillograms of the advertisement call of *Vitreorana micaelae* sp. nov. **A**. Call type I, short advertisement call, formed by one note of type A; **B**. Three-dimensional aspect of call type I, note type A; **C**. Call type II, long advertisement call showing four notes, being one note type A and three notes type B; **D**. Three-dimensional aspect of call type II. The calls were gathered from three individuals recorded at the type locality, municipality of Luziânia, state of Goiás, Brazil. Recorded on 05 December 2014; 21:32h. Air temperature 20 °C, relative air humidity 86%.

The advertisement call of *Vitreorana micaelae* sp. nov., is composed of two call types: a short call, named here type I (Fig. 4A, B) and a long call, named type II (Fig. 4C, D). The parameters of each call can be visualized in Table 2. The alternation between the emission of the short and long calls apparently does not follow a defined pattern. Call type I is as a single note (note A) (*n* = 10), structured with two or three pulses. Notes in short calls last 17.1–25.1 ms (22.5 ± 2.4 ms). Note A, besides its larger amplitude than the other type of note emitted by the new species, also presents discrete frequency modulation in its terminal portion (Fig. 4A). The dominant frequency ranges from 4651–4727 Hz. Call type II is multi-note call, composed of long calls in a combination of one note (note A) with two or three notes (each note here called note B) (*n* = 7) (Fig. 4C; example of a call type II composed of three notes type B). Each note B is also structured with 2–3 pulses. The long call duration ranges from 45.6–115.6 ms (83.8 ± 21.2 ms). In the calls type II, long calls, the first note (note A) has a progressive increase in amplitude until it reaches the second part, in which two or three type notes B are placed in sequentially and separated by brief gaps of silence (Fig. 4C, D). The dominant frequency ranges from 4694.2–4758.88 Hz.

**Table 2.** Acoustic measurements of the calls described the species in the genus *Vitreorana*. Means and standard deviations are followed by the ranges in parentheses. Parameters without minimum and maximum are given as single values, otherwise as mean ± standard deviation.

**Species**	**Emission pattern**	**Call Duration (ms)**	**Pulse number by notes**	**Note Rate (note/second)**	**Dominant frequency (Hz)**	**Reference**
*Vitreorana micaelae* sp. nov. (Luziânia, GO)	Call type I	22.5 ± 2.4 (17.1–25.1)	2–3	45.4 ± 5.5 (39,8–58.5)	4720.1 ± 36.3 (4651.2–4737.3)	This work
	Call type II	83.8 ± 21.2 (45.6–115.6)	2–3	44.4 ± 3.0 (41.1–47.4)	4718.2 ± 21.6 (4694.2–4758.8)	
*Vitreorana baliomma* (Murici, AL)	Single call	192 ± 4 (185–198)	7	36.5 ± 1 (35.3–37.8)	4979 ± 102 (4694–5081)	Bang et al. 2020
*Vitreorana eurygnatha* (São Gotardo, MG)	Single call	168 ± 20 (135–226)	3–4	19.7 ± 1.9 (15.9–24.4)	4796 ± 172 (4453–5016)	Bang et al. 2020
*Vitreorana eurygnatha* (Vargem Bonita, MG)	Single call	131 ± 5 (123–143)	3–4	23.5 ± 1.7 (22.4–28)	4523 ± 39 (4500–4593)	Bang et al. 2020
*Vitreorana eurygnatha* (Santa Teresa, ES)	Single call or sequence of 2–3 pulse clusters	216 ± 25 (189–249)	16–22	84 ± 4.5 (78.8–89.7)	4830 ± 96 (4694–4995)	Bang et al. 2020
*Vitreorana franciscana* (São Gotardo, MG)	Single call or sequence of 3–10 calls	52 ± 10 (33–73)	4–7	100.9 ± 8 (84.5–121.2)	4605 ± 91 (4359–4734)	Bang et al. 2020
*Vitreorana franciscana* (Vargem Bonita, MG)	Single call	56 ± 6 (48–69)	5–7	102.5 ± 9 (90.9–125)	4103 ± 39 (4031–4171)	Bang et al. 2020
*Vitreorana parvula* (Lages, SC)	Single call or a sequence of 2–9 calls	23.7 ± 6.6 (4.3–43.2)	1–5	134.6 ± 17.7 (62.3–232.6)	4583 ± 251 (4125–5063)	Zucchetti and Castroviejo-Fisher 2024
*Vitreorana parvula* (Florianópolis, SC)	Single call	28 ± 3.5 (21.2–35.2)	3–4	134.6 ± 7.7 (113.6–142.3)	4503 ± 114 (4406–4781)	Zucchetti and Castroviejo-Fisher 2024
*Vitreorana parvula* (Florianópolis, SC)	Single call or notes in pairs	37 ± 0 (20–84)	2–4	77.8 ± 4.4 (35.7–129)	4893 ± 57 (4651–4996)	Haga et al. 2014
*Vitreorana parvula* (Itapeva, MG)	Single call or notes in pairs	36 ± 1 (13–57)	1–5	97.6 ± 9.1 (41.6–230.7)	4592 ± 346 (3962–5063)	Haga et al. 2014
*Vitreorana parvula* (San Antonio, Missiones, Argentina)	Single call or a sequence of 2–9 notes	38 ± 13 (13–85)	1–5	128.5 ± 11 (91–166.7)	4642 ± 109 (4313–4875)	Zaracho 2014

#### Genetic data.

Genetic distances among *Vitreorana* species range from 3.5–9.6%, with the new species showing the lowest divergence from *V. parvula* (Table 3). Pairwise genetic distances between the new species and congeners are as follows: *V. parvula* (3.5–5.2%), *V. antisthenesi* (5.8%), *V. castroviejoi* (6.7%), *V. eurygnatha* (6.7%), *V. balinomma* (7.9%), *V. helenae* (8.0%), *V. gorzulae* (8.7%), and *V. ritae* (8.7%). The genetic distance between the two analyzed specimens of *V. parvula* (from the municipalities of Lages and Tangará, State of Santa Catarina, Brazil) is 0.4%.

**Table 3.** Uncorrected *p*-distances of partial 16S for nine species of *Vitreorana* species, including the new species. Numbers in front of species names refer to acronyms and museum numbers. Specimens of the species (*V. parvula*) less divergent (3.5–5.2%) are shown in bold. Distances are expressed in percentages.

	**1**	**2**	**3**	**4**	**5**	**6**	**7**	**8**	**9**	**10**	**11**
1. *Vitreorana micaelae* sp. nov. CEPB10157	0.00										
2. *Vitreorana micaelae* sp. nov. CEPB10154	0.00	0.00									
3. *Vitreorana parvula* CFBH15323	**3.51**	**3.51**	0.00								
4. *Vitreorana parvula* CFBH11709	**5.18**	**5.18**	3.60	0.00							
5. *Vitreorana antisthenesi* MHNLS17050	5.81	5.81	4.56	5.57	0.00						
6. *Vitreorana castroviejoi* MHNLS17289	6.75	6.75	5.00	6.40	3.54	0.00					
7. *Vitreorana gorzulae* MHNLS17142	8.69	8.69	6.54	7.24	7.19	6.70	0.00				
8. *Vitreorana eurygnatha* CFBH5121	6.73	6.73	4.85	5.08	5.81	7.42	7.93	0.00			
9. *Vitreorana baliomma* MCP14120	7.88	7.88	6.25	8.12	4.44	4.92	7.43	9.37	0.00		
10. *Vitreorana helenae* MHNLS17139	8.00	8.00	6.35	7.62	7.05	5.76	7.64	9.58	7.69	0.00	
11. *Vitreorana ritae* MB165INPA31273	8.67	8.67	7.48	8.93	7.69	7.03	8.66	8.66	9.02	3.39	0.00

#### Etymology.

The specific epithet honors the biologist Micaela de Jolepian L. Vaz, the beloved wife of the first author, in recognition of her unwavering support for his research on Neotropical biodiversity.

#### Natural history and distribution remarks.

**Figure 5.**
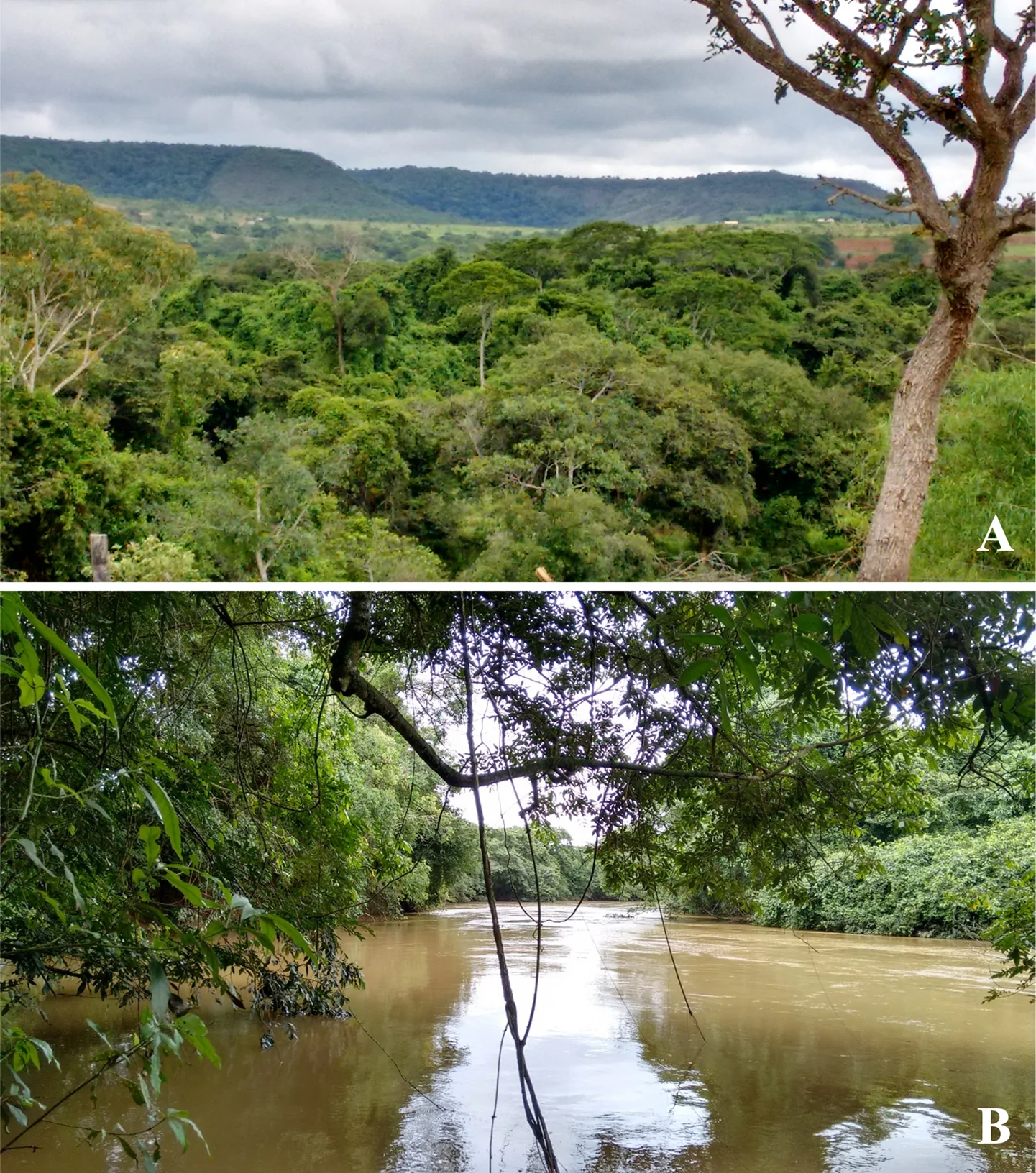
Type locality of *Vitreorana micaelae* sp. nov. **A**. Semidecidual Forest in contact with Gallery and Ciliary Forests of São Bartolomeu River; **B**. Bank on São Bartolomeu River. Photos by C. F. Rocha.

**Figure 6.**
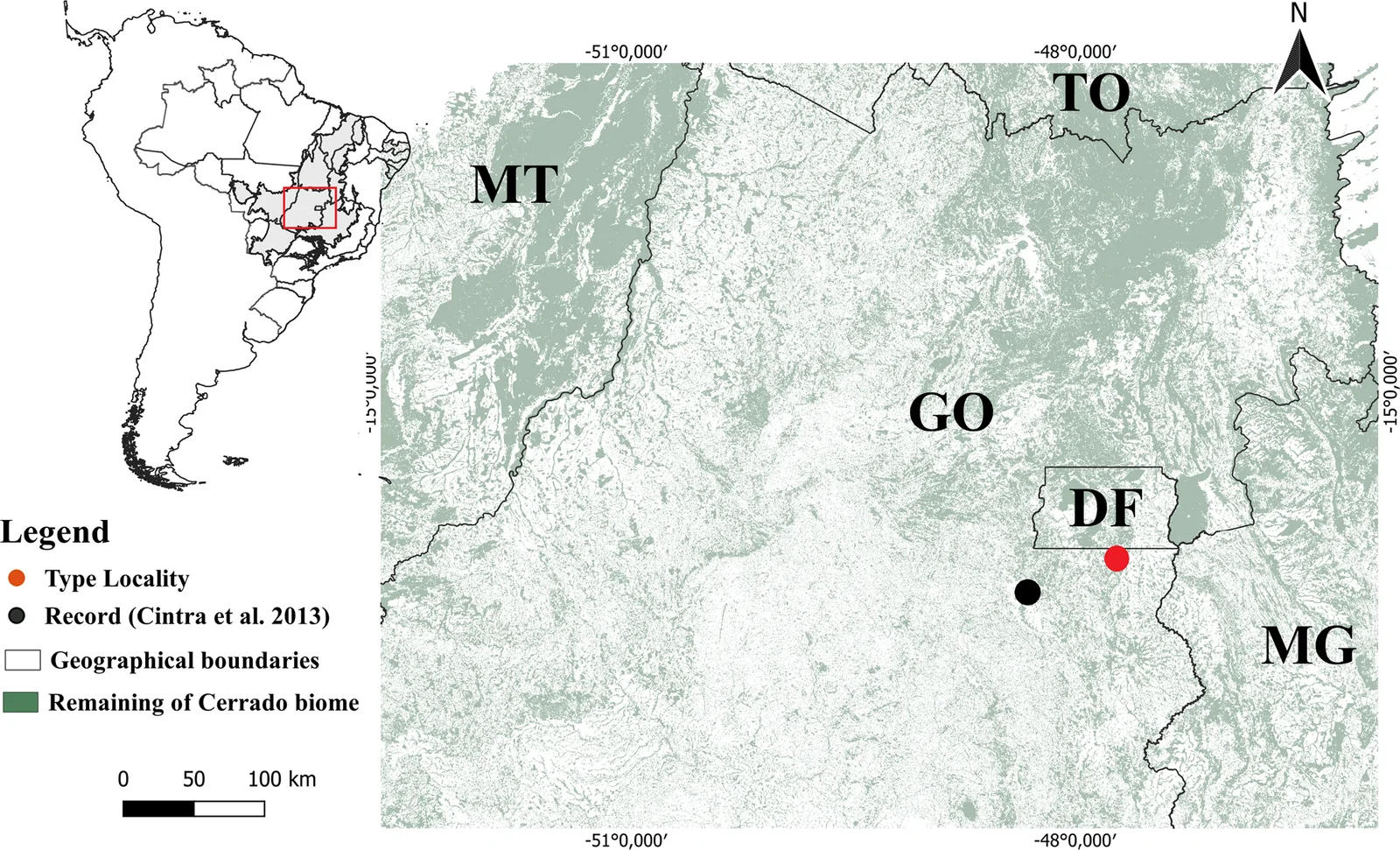
Map showing the type locality of the *Vitreorana micaelae* sp. nov. (red circle), Luziânia municipality, Brazil, and the register by Cintra et al. (2013) (CEPB 7712; black circle). DF = Federal District; GO = state of Goiás; TO = state of Tocantins; MT – state of Mato Grosso; MG = state of Minas Gerais.

*Vitreorana micaelae* sp. nov. is a habitat-specialist species occurring in semideciduous forest in ecotonal contact with riparian and gallery forests, along small streams (Fig. 5). The new species is known from two localities in the municipality of Luziânia, state of Goiás, central Brazil: the type locality and the record reported by Cintra et al. (2013) (CEPB 7712) (16°21'29"S, 48°18'22"W). The type locality is a gallery forest along a small stream near the forested bank of the São Bartolomeu River (Fig. 6), a tributary of the Paranaíba River basin. Calling males were observed ~ 2.5 m above the stream water on vegetation along the stream banks. Additional natural history data are unavailable.

## Discussion

*Vitreorana micaelae* sp. nov. is the eleventh described species of the genus *Vitreorana*, being morphologically most similar to *V. eurygnatha*, by sharing rounded and weakly pigmented cloacal tubercles, a non-pigmented tympanum, minute melanophores regularly distributed over the dorsal surface of the head, body, and limbs. However, *Vitreorana micaelae* sp. nov. can be readily distinguished by its rounded snout in dorsal view, being truncate in *V. eurygnatha*; snout profile sloping (truncated or rounded in *V. eurygnatha*), shagreened dorsum (smooth in *V. eurygnatha*), and the characteristics of the advertisement call. Also, the new species showed lowest divergence from *V. parvula* (3.5–5.2%) than to *V. eurygnatha* (6.7%).

The new species represents the second *Vitreorana* species endemic to the Brazilian Cerrado and the first to the Goiás state. *Vitreorana franciscana*, described a decade ago, occurs in Serra da Canastra National Park and in the municipality of Presidente Olegário, State of Minas Gerais (Santana et al. 2015; Maffei et al. 2024). In contrast, *Vitreorana micaelae* sp. nov. is the only species of the genus currently known from the core area of the Cerrado.

Considering the distribution and complexity of forested habitats within the Cerrado, as well as the occurrence of two additional species in gallery forests in transitional zones between the Cerrado and Atlantic Forest (*V. eurygnatha* and *V. franciscana*), the diversity of *Vitreorana* in the Cerrado is likely underestimated (Paz et al. 2019; Bang et al. 2020). We therefore recommend increasing the sampling in poorly-surveyed areas and further taxonomic and biogeographical studies to better assess species richness and clarify phylogeographic relationships among lineages of *Vitreorana* in the Cerrado and Atlantic Forest. Ideally, such studies should integrate morphological and bioacoustic data with expanded molecular datasets, including additional mitochondrial and nuclear markers, as well as genomic approaches.

The genus *Vitreorana* is composed of species typically related to forested environments, inhabiting riverine canopy vegetation near permanent streams and creeks (Santana et al. 2015; Maffei et al. 2024). Almost all species occur in the Amazonia and the Atlantic Rainforests (Frost 2026), being related to umbrophilous formations (Santana et al. 2015; Maffei et al. 2024). Along with *Vitreorana franciscana* and *V. eurygnatha*, *V. micaelae* sp. nov. represents the third species associated with humid umbrophilous forests in the Cerrado, such as gallery and ciliary forests. The potential closer relationship between *V. micaelae* sp. nov. and species related to the Atlantic Forest, suggests a historical relationship between some gallery forests in the Cerrado and forested habitats with Atlantic Forests (Redford and Fonseca 1986; Meave et al. 1991), affecting the presence of Atlantic lineages of frogs in the Cerrado core area (Brandão and Araujo 2001; Valdujo et al. 2012; Valdujo et al. 2013; Azevedo et al. 2016) and determining certain aspects of endemicity in Cerrado frogs (Valdujo et al. 2013; Berneck et al. 2017).

The primary anthropogenic threats to the species include the construction of river dams, land conversion for agriculture, which can lead to degradation of rivers and streams within its range, and potential mining activities in the region. Due to the insufficient information available to assess population fragmentation and the impact of these threats on the species, we recommend that *Vitreorana micaelae* sp. nov. should be classified as Data Deficient (DD) according to IUCN criteria (IUCN 2025). This matter may also be influenced by the greater research attention traditionally given to the habitats where *Vitreorana* species occur. Historically, the open phytophysiognomies of the Cerrado have been more extensively surveyed than forested formations (Bertoluci et al. 2007; Ramalho et al. 2018), contributing to an uneven distribution of knowledge and potentially underestimating the diversity of lineages such as Centrolenidae. Consequently, many forest-associated anuran lineages remain poorly represented in museum collections and molecular datasets, limiting our understanding of their taxonomy, ecology, and evolutionary history (Hortal et al. 2015).

## Supplementary Material

XML Treatment for Vitreorana micaelae

## Additional information

### Artificial Intelligence (AI) use

The authors accept full responsibility for the content of the manuscript, including the disclosure of any use of AI.

ChatGPT was used to improve style, correct grammar, and wording. All sentences were originally written and revised by the authors.

### Author ORCIDs

W. Vaz-Silva https://orcid.org/0000-0001-6235-5331

S. Pereira de Andrade https://orcid.org/0000-0003-3078-9375

R.A. Brandão https://orcid.org/0000-0003-3940-2544

N.M. Maciel https://orcid.org/0000-0001-5654-0645

### Data availability

All of the data that support the findings of this study are available in the main text or Supplementary Information.

## Supplementary materials

Supplementary material 1 Analyzed specimens Data type doc https://binary.pensoft.net/file/1763910This dataset is made available under the Open Database License (http://opendatacommons.org/licenses/odbl/1.0/). The Open Database License (ODbL) is a license agreement intended to allow users to freely share, modify, and use this Dataset while maintaining this same freedom for others, provided that the original source and author(s) are credited. Wilian Vaz-Silva, Sheila Pereira de Andrade, Cezar Filho Rocha, Edmar Pereira Victor Jr., Reuber Albuquerque Brandão, Natan Medeiros Maciel

Supplementary material 2 Species names, localities, GenBank accession numbers, and references of species and specimens of *Vitreorana* used to obtain the genetic distances Data type doc https://binary.pensoft.net/file/1763911This dataset is made available under the Open Database License (http://opendatacommons.org/licenses/odbl/1.0/). The Open Database License (ODbL) is a license agreement intended to allow users to freely share, modify, and use this Dataset while maintaining this same freedom for others, provided that the original source and author(s) are credited. Wilian Vaz-Silva, Sheila Pereira de Andrade, Cezar Filho Rocha, Edmar Pereira Victor Jr., Reuber Albuquerque Brandão, Natan Medeiros Maciel
