# Supplementary material for: Unveiling a small new glassfrog of the genus Vitreorana (Anura, Centrolenidae) from Cerrado, central Brazil
Source: Zookeys. 2026 Sep 3;1291:93–110. doi: 10.3897/zookeys.1291.193210 (PMC13563115; doi:10.3897/zookeys.1291.193210)
Supplement: Supplementary file 1 [file zookeys-1291-093_article-193210__-s001.doc]

**Supplementary File S1. Analyzed specimens.**

***Vitreorana eurygnatha***.— BRAZIL: Minas Gerais state: Mariana: MNRJ 30606 and 30607; Itamonte: MNRJ 19384; Lima Duarte: MNRJ 24663 and 24664; Cristália: MNRJ 40216; São Paulo state: Ribeirão Branco: MNRJ 17649; Rio de Janeiro state: Teresópolis: MNRJ 32872; Rio de Janeiro: MNRJ 73128–73134. Goiás state: Luziânia: CEPB 7712. ***Vitreorana baliomma***.—BRAZIL: Bahia state: Itamaraju: MNRJ 46857 (holotype). ***Vitreorana franciscana***.—BRAZIL: Minas Gerais state: São Roque de Minas: Parque Nacional da Serra da Canastra: MNRJ 81776 (holotype), 81777 and 81779 (paratopotypes). ***Vitreorana parvula***.— BRAZIL: Minas Gerais state: Catas Altas: MNRJ 77778–77780; São Paulo state: Botucatu: MNRJ 32510 and 325111; Paranapicaíba: MNRJ 3650; Rio de Janeiro state: Tijuca: MNRJ 52702; Duque de Caxias: MNRJ 73012.
