# Supplementary material for: Unveiling a small new glassfrog of the genus Vitreorana (Anura, Centrolenidae) from Cerrado, central Brazil
Source: Zookeys. 2026 Sep 3;1291:93–110. doi: 10.3897/zookeys.1291.193210 (PMC13563115; doi:10.3897/zookeys.1291.193210)
Supplement: Supplementary file 2 [file zookeys-1291-093_article-193210__-s002.doc]

**Supplementary File S2. Species names, localities, GenBank accession numbers, and references of species and specimens of *Vitreorana* used to obtain the genetic distances.**

***Vitreorana antisthenesi***.—Venezuela: Estado Aragua: Parque Nacional Henri Pittier, Estación Biológica Rancho Grande, EU447287, Castroviejo-Fisher et al. (2008). ***Vitreorana balinomma***.—Brazil: Estado da Bahia: RPPN Ararauna, Una, MW366914, Zucchetti et al. (2021). ***Vitreorana castroviejoi***.—Venezuela: Estado Sucre: Cerro Humo, EU663004, Guayasamin et al. (2008). ***Vitreorana eurygnatha***.—Brazil: Estado de São Paulo: Campos do Jordão, KU495607, Lyra et al. (2016). ***Vitreorana gorzulae***.—Venezuela: Estado Bolívar: km 127 on the El Dorado-Santa Elena de Uairén road, EU266749, Kok et al. (2008). ***Vitreorana helenae*.**—Venezuela: Estado Bolivar: Salto Karuay, EU663012, Guayasamin et al. (2008). ***Vitreorana micaelae*** sp. nov.—Brazil: Estado de Goiás: Luziânia, PV961221 and PV961222, This work. ***Vitreorana parvula***.—Brazil: Estado de Santa Catarina: Lages, MH206244, de Resende et al. (unpublished). ***Vitreorana parvula***.—Brazil: Estado de Santa Catarina: Tangará, KU495615, Lyra et al. (2016). ***Vitreorana ritae***.—Brazil: Estado do Amazonas: Tamaga, EU663202, Guayasamin et al. (2008).
